# Comparative evaluation of two NGS-based assays for somatic hypermutation analysis of IGHV genes in chronic lymphocytic leukemia

**DOI:** 10.1007/s44313-026-00132-7

**Published:** 2026-04-06

**Authors:** Yehyun Kang, Hyeonah Lee, Yu Jin Park, Seung-Tae Lee, Jong Rak Choi, Saeam Shin

**Affiliations:** 1 Graduate School of Medical Science, Brain Korea 21 PLUS Project, Yonsei University College of Medicine, Seoul, Republic of Korea; 2 Division of Zoonotic and Vector-Borne Disease Research, Center for Infectious Disease Research, Korea National Institute of Health, Cheongju, Republic of Korea; 3 Department of Laboratory Medicine, Yonsei University College of Medicine, Severance Hospital, 50-1 Yonsei-Ro, Seodaemun-Gu, Seoul, 03722 Republic of Korea; 4 Dxome Co., Ltd., Seongnam-Si, Gyeonggi-Do, Republic of Korea

**Keywords:** Chronic lymphocytic leukemia, Somatic hypermutation, Sanger sequencing, Next-generation sequencing, Immunoglobulin heavy chain gene

## Abstract

**Purpose:**

Somatic hypermutation (SHM) of the immunoglobulin heavy chain variable (*IGHV*) region is a key prognostic marker in chronic lymphocytic leukemia (CLL). Traditionally, SHM status has been determined using Sanger sequencing (SS); however, next-generation sequencing (NGS) provides an alternative method for assessing both SHM status and clonal rearrangements. This study aimed to compare the performance of two commercially available NGS assays for evaluating *IGH* clonality and SHM status in CLL.

**Methods:**

In this retrospective study, 42 samples from patients diagnosed with CLL were analyzed. Genomic DNA extracted from peripheral blood or bone marrow aspirates was analyzed using two commercial NGS assays: the LymphoTrack® *IGHV* Leader (Leader) and *IGH* FR1 (FR1) assays (Invivoscribe, CA, USA). SS was performed as the reference method for SHM assessment.

**Results:**

The Leader assay identified clonality in 95.2% of cases, whereas the FR1 assay detected clonality in 88.1%. Conclusive SHM status was determined in 90.5% of samples using the Leader assay and in 76.2% using the FR1 assay; when results from both assays were combined, the rate increased to 92.9%. Among samples with conclusive results by both SS and each NGS assay, the Leader assay demonstrated higher concordance with SS (97.1%, 34/35) than the FR1 assay (86.2%, 25/29). Greater variability in clonal detection was observed with the FR1 assay.

**Conclusion:**

These findings indicate that the Leader assay provides a more reliable assessment of SHM status, with higher concordance with SS. Although the FR1 assay may offer additional information regarding clonal patterns, its results should be interpreted cautiously. Given the limited sample size, further studies are warranted to validate these findings. Overall, the Leader assay appears to be more suitable as a primary tool for SHM evaluation, with FR1 results serving a complementary role when interpreted in clinical context.

**Supplementary Information:**

The online version contains supplementary material available at 10.1007/s44313-026-00132-7.

## Introduction

Lymphoid cells are distinct from other somatic cells because they undergo somatic gene rearrangement of their antigen receptor genes during development [1]. Because leukemias and lymphomas arise from the malignant transformation of individual lymphoid cells, neoplastic cells in affected patients generally exhibit one or more clonal antigen receptor gene rearrangements. Therefore, assays that detect clonal rearrangements of immunoglobulin (IG) receptors are useful for investigating lymphoid malignancies [2].

In addition, the hypermutation status of the immunoglobulin heavy chain variable (*IGHV*) gene provides important prognostic information for patients with chronic lymphocytic leukemia (CLL) [3]. Somatic hypermutation (SHM) is defined as a sequence difference of 2% or more from the germline variable domain sequence, whereas a difference of less than 2% indicates the absence of SHM [4]. An inconclusive result refers to cases in which no clonal sequence is identified, sequencing data are insufficient, clonality cannot be demonstrated, or discordant dual rearrangements are present. The presence of SHM is associated with a favorable prognosis in CLL, whereas its absence is associated with a poor prognosis [5].

Traditionally, Sanger sequencing (SS) has been considered the gold standard for SHM testing. However, recent advances in next-generation sequencing (NGS) have addressed several limitations of SS. One major limitation of SS is the extensive diversity of IG gene rearrangements, which requires complex multiplex polymerase chain reaction (PCR) amplification. In addition, because SS is a low-throughput method, it has limited sensitivity for detecting minor subclones and intraclonal diversification. In contrast, NGS enables the simultaneous assessment of *IGHV* SHM status and mutation profiles with higher sensitivity and throughput [6].

We compared two commercially available NGS-based assays for clonality and SHM determination: the LymphoTrack® *IGH* FR1 Assay (FR1 assay) (Invivoscribe, San Diego, CA, USA) and the LymphoTrack® *IGHV* Leader SHM Assay (Leader assay) (Invivoscribe). Both assays utilize a single multiplex master mix to detect clonal *IGH* gene rearrangements; however, they differ in their target regions [7]. The Leader assay targets the leader and joining regions of the *IGH* gene, whereas the FR1 assay targets the conserved framework region 1 (FR1) within the variable region as well as the joining region of the *IGH* gene. Both assays enable the detection of clonality and identification of clonal rearrangement sequences using a single multiplex master mix, thereby allowing assessment of SHM status and tracking of the same clonal sequence in subsequent samples. These assays were developed for complementary use based on differences in their target regions and analytical objectives. When the SHM status determined by the FR1 assay is close to the cutoff value, or when the Leader assay fails to detect a clonal sequence, use of the alternative assay is recommended to clarify the SHM result.

Because performing both assays requires additional costs and laboratory resources, clinical laboratories may elect to perform only one assay or adopt a stepwise approach in which the second assay is performed only when the initial result is inconclusive. Therefore, a clear understanding of the concordance and performance characteristics of these two assays is needed. In this context, this study aimed to compare the two NGS assays with respect to clonality detection and SHM determination.

## Materials and methods

### Samples and NGS

Peripheral blood or bone marrow samples obtained at diagnosis from 42 patients with CLL were included in this study. All samples were retrospectively selected from patients who had undergone routine diagnostic evaluation. This study, which evaluated testing methods using residual clinical samples, was exempt from ethical review by the Institutional Review Board of Severance Hospital (IRB No. 4–2024-0638). The study was conducted at Severance Hospital, Seoul, Republic of Korea, and included samples collected between August 2024 and June 2025.

Genomic DNA was extracted from peripheral blood or bone marrow aspirates using the QIAamp DNA Mini Kit (Qiagen, Hilden, Germany). SS of the *IGHV* gene was performed in all 42 cases using *IGHV* leader primers, as recommended by the European Research Initiative on CLL guidelines, and served as the reference method for SHM assessment [8, 9]. FASTA files generated by SS were analyzed using IMGT/V-QUEST (IMGT®, the international ImMunoGeneTics information system®, http://www.imgt.org) and IgBLAST (National Center for Biotechnology Information, http://www.ncbi.nlm.nih.gov/igblast). Cases in which SHM status could not be determined by SS because of insufficient sequence length, low-quality reads, or ambiguous alignment were classified as inconclusive.

For NGS, PCR amplicons were purified using AMPure XP beads (Beckman Coulter Inc., Brea, CA, USA) to remove excess primers, nucleotides, salts, and enzymes. Both the FR1 and Leader assays were sequenced on the MiSeqDx platform (Illumina, San Diego, CA, USA) using the MiSeq Reagent Kit v2 (500 cycles) for the FR1 assay and the MiSeq Reagent Kit v3 (600 cycles) for the Leader assay.

The Leader assay targets the full-length *IGHV* variable region, allowing a more accurate assessment of SHM, whereas the FR1 assay targets a partial variable (V) region and may be affected by primer-binding bias depending on *IGHV* gene usage. Sequence data from both assays were analyzed using LymphoTrack® Software–MiSeq v2.4.3 (Invivoscribe Technologies) for clonality assessment and SHM interpretation according to the manufacturer’s instructions.

### SHM and clonality assessment

SHM status was classified using a 98% *IGHV* sequence identity threshold. Sequences showing < 98% identity were categorized as mutated *IGHV*, whereas those with ≥ 98% identity were classified as unmutated *IGHV* [10]. SHM status was considered inconclusive when reliable classification was not possible because of ambiguous V-gene alignment, insufficient evaluable V-gene sequence length, or unproductive rearrangements, in accordance with the manufacturer’s interpretation guidelines. Clonality was assessed according to the manufacturer’s interpretation criteria based on the relative abundance of merged *IGHV* sequences. Evidence of clonality was defined as the presence of a dominant merged sequence exceeding a predefined read percentage threshold relative to the third most frequent sequence, with thresholds applied according to the total read count. Samples that did not meet these criteria were classified as showing no evidence of clonality. Cases that did not fulfill the criteria for either clonal or non-clonal classification were categorized as inconclusive. Inconclusive SHM and clonality results were retained as separate categories in downstream analyses.

### Statistical analysis

Statistical analyses were performed using R software (version 4.3.1; R Foundation for Statistical Computing, Vienna, Austria). Categorical variables, including clonality detection rates and the proportion of samples with conclusive SHM results, were compared between assays using McNemar’s exact test. Continuous variables, including clone size and mutation rate (%), were compared using the Wilcoxon signed-rank test. Agreement in SHM classification between each NGS assay and SS was assessed using Cohen’s kappa coefficient and interpreted according to conventional criteria. Correlations between mutation rates obtained by SS and each NGS assay were evaluated using Pearson’s correlation coefficient with corresponding 95% confidence intervals. A two-sided *p*-value < 0.05 was considered statistically significant.

## Results

### Sequencing quality

Sequencing quality was assessed using total read counts and index read quality metrics. The median number of reads per sample was 375,884 (interquartile range [IQR], 174,547–542,439) for the *IGHV* Leader assay and 387,082 (IQR, 303,525–526,487) for the *IGH* FR1 assay. Except for one polyclonal case identified by the Leader assay, samples classified as showing no evidence of clonality reflected a true absence of detectable clones rather than insufficient sequencing depth.

### Clonality detection

**Fig. 1:**
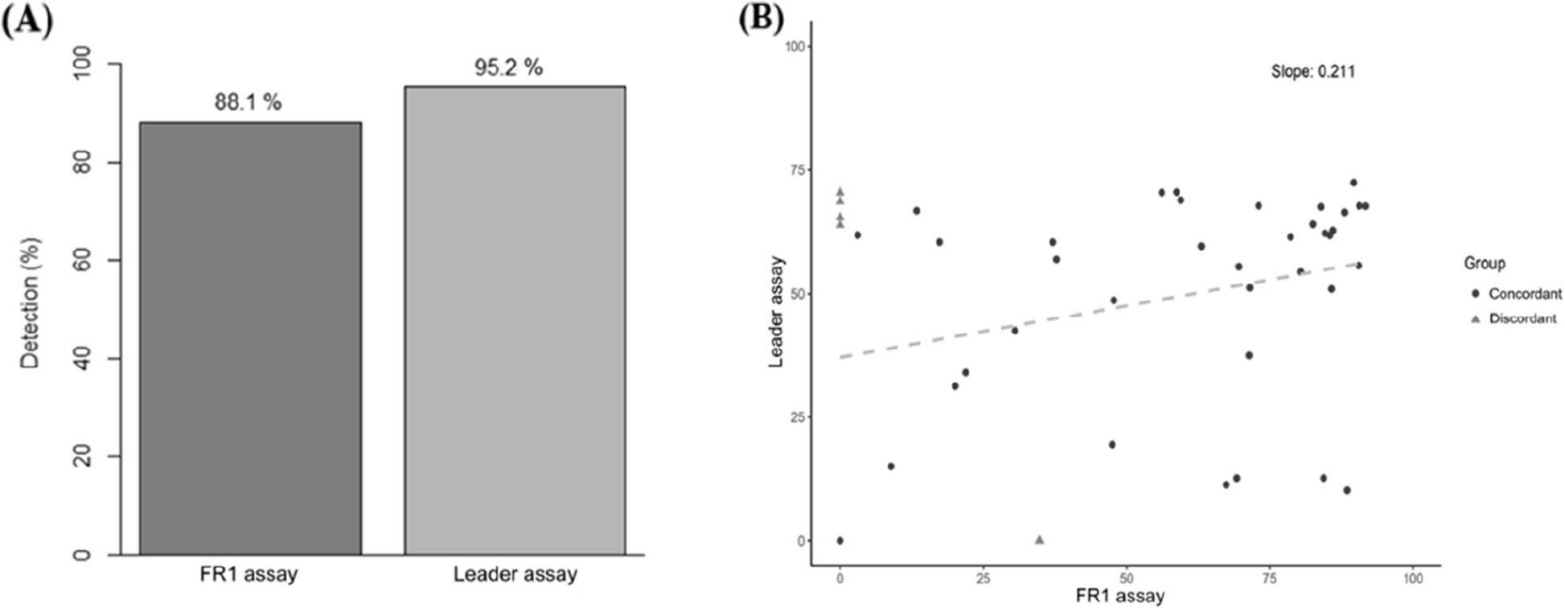
Comparison of clonality detection between the FR1 and Leader assays. **A** Bar graph showing clonality detection rates for the FR1 and Leader assays. **B** Scatter plot comparing the sizes of matched clones detected by the two assays. Abbreviation: *IGHV*, immunoglobulin heavy chain variable region

For clonality detection, 37 samples (88.1%) showed identical results between the Leader and FR1 assays (Table 1). In the Leader assay, 40 of 42 samples (95.2%) showed evidence of clonality, whereas 37 of 42 samples (88.1%) in the FR1 assay showed evidence of clonality (Fig. 1A). Notably, four samples that demonstrated clonal rearrangements in the Leader assay were classified as showing no evidence of clonality in the FR1 assay, indicating a higher likelihood of clone underdetection with the FR1 assay. Although the Leader assay identified clonality in a slightly greater number of cases, this difference was not statistically significant (exact McNemar’s test, *p* = 0.375).

**Table 1:** Concordance of clonality detection between the leader and FR1 assays

Clonality		Leader assay	
		Positive	Negative
FR1 assay	Positive	36	1
	Negative	4	1

Among samples with clonality detected by both assays, 23 (63.9%) exhibited higher clonality values in the FR1 assay than in the Leader assay (Fig. 1B). However, overall comparison of clone sizes between the two assays showed no statistically significant difference (Wilcoxon signed-rank test, *p* = 0.228), indicating comparable clonality measurements across samples.

### SHM determination

For SHM determination, the two NGS assays showed concordant results in 71.4% (30/42) of samples (Table 2; Tables S1). The Leader assay yielded inconclusive results in 9.5% (4/42) of samples, whereas the FR1 assay yielded inconclusive results in 23.8% (10/42).

**Table 2:** Somatic hypermutation classification results obtained by the leader and FR1 assays

Somatic hypermutation		Leader assay		
		Mutated SHM	Unmutated SHM	Inconclusive
FR1 assay	Mutated SHM	22	0	1
	Unmutated SHM	4	5	0
	Inconclusive	7	0	3

*Abbreviation*: *SHM* somatic hypermutation

Among the four samples with inconclusive results by the Leader assay, one (25%) was classified as mutated by the FR1 assay. Conversely, among the 10 samples with inconclusive results by the FR1 assay, seven (70%) were classified as mutated by the Leader assay.

When the results of both assays were combined, the proportion of samples with inconclusive SHM status decreased to 7.1% (3/42). Although the Leader assay tended to yield fewer inconclusive results than the FR1 assay, this difference did not reach statistical significance (exact McNemar’s test, *p* = 0.07).

Among samples with conclusive SHM results by either assay, 71.9% (23/32) were classified as mutated *IGHV* by the FR1 assay, whereas 86.8% (33/38) were classified as mutated *IGHV* by the Leader assay. However, the difference in SHM detection rates between the assays did not reach statistical significance (exact McNemar’s test, *p* = 0.125).

**Fig. 2:**
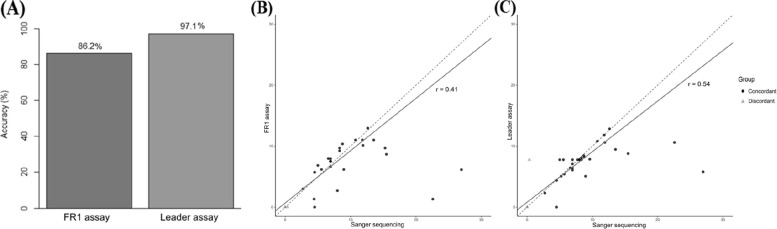
Somatic hypermutation (SHM) detection accuracy and mutation rate concordance across assays. **A** Bar plot showing SHM classification accuracy of the FR1 and Leader assays compared with Sanger sequencing. **B** Scatter plot comparing mutation rates (%) in the analyzed partial V-gene region between Sanger sequencing and the FR1 assay. **C** Scatter plot comparing mutation rates (%) in the analyzed partial V-gene region between Sanger sequencing and the Leader assay. Abbreviations: *IGHV*, immunoglobulin heavy chain variable region; *SHM*, somatic hypermutation

The SHM detection performance of both assays was further evaluated using SS as the reference method (Tables S2 and S3). An inconclusive SHM result was observed in 9.5% (4/42) of samples by SS. Among samples with conclusive results by both SS and each NGS assay, the Leader assay showed higher concordance with SS (97.1%, 34/35) than the FR1 assay (86.2%, 25/29) (Fig. 2A).

To further quantify agreement, Cohen’s kappa coefficient was calculated for each assay relative to SS. The Leader assay demonstrated almost perfect agreement (kappa = 0.842, *p* < 0.001), whereas the FR1 assay showed fair agreement (kappa = 0.247, *p* = 0.027). Figures 2B and C display scatter plots comparing mutation rates (%) in the partial V region between SS and each assay (Fig. 2B, C). The Leader assay showed a moderate positive correlation with SS (Pearson’s r = 0.544, 95% confidence interval [CI], 0.241–0.750, *p* = 0.0013), whereas the FR1 assay showed a weaker positive correlation (Pearson’s r = 0.413, 95% CI, 0.047–0.681, *p* = 0.029). These findings indicate that the Leader assay provides more reliable and consistent SHM assessment than the FR1 assay, particularly in samples with low-level or borderline SHM.

## Discussion

In this study, both NGS assays demonstrated a high rate of successful clonal sequence detection in diagnostic CLL samples, and SHM status was determined in a large proportion of cases. Together, the Leader and FR1 assays provided complementary information for SHM assessment, particularly in cases with borderline or inconclusive results using conventional methods. Previous studies have similarly reported that the combined use of the Leader and FR1 assays improves SHM detection efficiency [11].

Although prior reports have described comparable SHM detection rates between the two assays, the present study demonstrated a modestly lower SHM determination rate with the FR1 assay than with the Leader assay. This difference is more likely attributable to intrinsic assay characteristics rather than biological variation. The Leader assay uses primers spanning the full-length *IGHV* region, allowing more comprehensive coverage of SHM hotspots and facilitating more accurate sequence alignment. In contrast, the FR1 assay targets a shorter region within FR1, where primer-binding efficiency may be affected by sequence variability, potentially leading to underestimation of SHM or inconclusive results.

Although the proportion of mutated IGHV cases in our cohort (54.7% by the FR1 assay and 78.6% by the Leader assay) was comparable to that reported in previous studies of Korean patients with CLL, this finding should not be interpreted as evidence of population-specific biological differences. Rather, the observed variability in SHM determination is likely attributable to technical factors, including differences in primer design and coverage of SHM hotspots between the assays. In particular, the FR1 assay targets a more limited region of the IGHV gene, which may reduce its sensitivity for detecting IGHV mutations compared with the Leader assay. These findings suggest that the observed discrepancies in SHM status are more likely driven by assay-related characteristics than by ethnicity-related biological differences.

Given that the 2% *IGHV* mutation cutoff represents a critical clinical decision point, particular attention is warranted for cases near this threshold. In our cohort, only a small number of discordant cases were located close to the cutoff. Two cases showed concordant classification between the Leader and FR1 assays, including one unmutated case (1.35% in the Leader assay vs. 1.76% in the FR1 assay, based on mutation rates calculated over the respective analyzed V-gene regions) and one mutated case (2.32% in the Leader assay vs. 3.0% in the FR1 assay), suggesting overall agreement between the assays near the decision threshold. However, one case classified as mutated by the Leader assay (9.9%) showed discordant V-gene identities in the FR1 assay (10.96% and 1.76%), resulting in an inconclusive interpretation. This finding indicates that the presence of multiple rearrangements, particularly near the cutoff, may complicate SHM interpretation with the FR1 assay. In addition, one case was deemed inconclusive by the Leader assay because of failure to detect a clonal sequence but was classified as mutated by the FR1 assay, illustrating the complementary roles of the two assays in resolving interpretive uncertainty. Taken together, these observations suggest that reflex testing with an alternative assay may be considered when SHM results are borderline or inconclusive; however, further validation in larger cohorts is warranted. All discordant cases were supported by sufficient sequencing depth, suggesting that these discrepancies were unlikely to be attributable to sequencing quality.

Regarding clonality assessment, the FR1 assay tended to identify larger dominant clones and showed a higher frequency of undetected clonality than the Leader assay. This finding suggests a potential bias toward the detection of higher-abundance clones with FR1 primers, whereas smaller or subclonal populations may be underrepresented. Consequently, although the FR1 assay is suitable for clonality screening and may be advantageous for detecting dominant clones, the Leader assay appears to be more reliable for comprehensive clonal characterization.

Several limitations of this study should be acknowledged. First, the reference standard used for comparison—SS performed with *IGHV* leader primers—targets the same genomic region as the Leader assay, whereas the FR1 assay targets a distinct and shorter region of the *IGHV* gene. This design characteristic should be considered when interpreting the observed performance differences, particularly the higher concordance between the Leader assay and SS. Accordingly, these findings should be interpreted as reflecting relative performance within this reference framework rather than demonstrating clear superiority of one assay over the other. Second, the cohort size was relatively small (n = 42) and derived from a single institution, which may have reduced the statistical power to detect subtle differences between assays and limited generalizability. Third, technical differences in primer design and amplification efficiency between the assays may have influenced both clonality and SHM assessment. In addition, functional validation of clonal populations was not performed, limiting direct biological interpretation of the detected clonal rearrangements. Independent validation of discordant cases using methodologically distinct approaches would help elucidate the sources of disagreement between the assays. Such validation was not feasible within the design of the present study and therefore warrants further investigation. Future studies incorporating larger, multicenter cohorts and integrating IG light-chain (*IGK*/*IGL*) SHM analysis—recently suggested to have additional prognostic value—may further refine molecular risk stratification in CLL. In addition, longitudinal analysis of subclonal dynamics using NGS-based approaches may provide insights into clonal evolution and disease progression, thereby supporting more precise clinical monitoring.

## Conclusion

In conclusion, the FR1 assay appears to be suitable for clonality assessment in diagnostic CLL samples, whereas the Leader assay provides a more reliable evaluation of *IGHV* SHM status. Based on the observed differences in clonal representation, the FR1 assay may have potential utility in settings such as minimal residual disease monitoring; however, this application remains to be validated in longitudinal studies. The complementary strengths of these two assays suggest that their combined use may provide a more comprehensive molecular characterization of CLL.

## Supplementary Information

Supplementary Material 1. Table S1. Summary of clonality and SHM status assessed by the IGHV Leader assay, the IGHV FR1 assay, and Sanger sequencing in all patients. Table S2. Comparison of somatic hypermutation results between Sanger sequencing and the FR1 assay. Table S3. Comparison of somatic hypermutation results between Sanger sequencing and the Leader assay.

## Data Availability

Data are available upon reasonable request from the authors.
