# Supplementary material for: Comparative evaluation of two NGS-based assays for somatic hypermutation analysis of IGHV genes in chronic lymphocytic leukemia
Source: Blood Res. 2026 Apr 6;61(1):14. doi: 10.1007/s44313-026-00132-7 (PMC13057106; doi:10.1007/s44313-026-00132-7)
Supplement: Supplementary file 1 — Supplementary Material 1. Table S1. Summary of clonality and SHM status assessed by the IGHV Leader assay, the IGHV FR1 assay, and Sanger sequencing in all patients. Table S2. Comparison of somatic hypermutation results between Sanger sequencing and the FR1 assay. Table S3. Comparison of somatic hypermutation results between Sanger sequencing and the Leader assay. [file 44313_2026_132_MOESM1_ESM.docx]

Table S1. Summary of clonality and SHM status assessed by IGHV Leader, IGHV FR1, and Sanger sequencing in all patients

|  | *IGHV* Leader assay | | | | *IGH* FR1 assay | | | | SS |
| --- | --- | --- | --- | --- | --- | --- | --- | --- | --- |
|  | Clonality | | SHM | | Clonality | | SHM | | SHM |
| ID | (%) | Status | V-gene mutated  rate (%) | Status | (%) | Status | V-gene  mutated  rate (%) | Status | Status |
| UPN1 | 70.52 | Mono | 4.39 | M | 58.74 | Mono | 5.73 | M | M |
| UPN2 | 67.77 | Mono | 0 | U | 73.05 | Mono | 0 | U | U |
| UPN3 | 19.43 | Mono | 7.77 | M | 47.5 | Oligo | 6.17 / 0.00 | I | M |
| UPN4 | 10.19 | Mono | 7.77 | M | 88.54 | Mono | 0 | U | I |
| UPN5 | 42.43 | Oligo | 9.9 | M | 30.52 | Oligo | 10.96 / 1.76 | I | M |
| UPN6 | 11.31 | Oligo | 7.77 | M | 67.38 | Mono | 6.84 | M | M |
| UPN7 | 55.7 | Mono | 11.82 | M | 90.56 | Mono | 11.01 | M | M |
| UPN8 | 67.55 | Mono | 8.11 | M | 83.92 | Mono | 9.69 | M | M |
| UPN9 | 68.91 | Oligo | 7.77 | M | 59.46 | Oligo | 2.70 / 10.27 | M | M |
| UPN10 | 12.64 | Mono | 7.77 | M | 69.22 | Oligo | 7.93 / 7.49 | M | M |
| UPN11 | 67.69 | Mono | 1.35 | U | 91.7 | Mono | 1.76 | U | U |
| UPN12 | 31.22 | Mono | 9.46 | M | 20.09 | Oligo | 11.01 / 11.89 | M | M |
| UPN13 | 61.85 | Mono | 7.09 | M | 3.07 | Oligo | 0.00 / 7.52 | I | M |
| UPN14 | 33.96 | Mono | 7.77 | M | 21.91 | Mono | 0 | U | M |
| UPN15 | 12.65 | Oligo | 10.81 | M | 84.41 | Mono | 11.01 | U | M |
| UPN16 | 61.49 | Mono | 12.84 | M | 78.63 | Mono | 12.95 | M | M |
| UPN17 | ND | ND | - | I | ND | ND | - | I | I |
| UPN18 | 70.45 | Mono | 13.91 | I | ND | ND | - | I | M |
| UPN19 | 67.76 | Mono | 1.36 | I | 90.66 | Mono | 1.33 | I | M |
| UPN20 | 66.45 | Mono | 2.32 | M | 88.09 | Mono | 3 | M | M |
| UPN21 | 65.44 | Mono | 7.85 | M | ND | ND | - | I | M |
| UPN22 | 55.48 | Oligo | 6.42 | M | 69.58 | Oligo | 7.52 / 6.64 | M | M |
| UPN23 | 48.68 | Mono | 0 | U | 47.78 | Mono | 0 | U | M |
| UPN24 | 61.81 | Oligo | 0 | U | 85.52 | Mono | 0 | U | U |
| UPN25 | 66.79 | Mono | 7.77 | M | 13.34 | Oligo | 9.25 / 0.00 | I | M |
| UPN26 | 56.94 | Oligo | 10.6 | M | 37.79 | Oligo | 10.13 / 11.01 | M | M |
| UPN27 | ND | Poly | - | I | 34.85 | Oligo | 9.69 / 7.49 | M | M |
| UPN28 | 64.04 | Mono | 8.87 | M | 82.58 | Mono | 8.77 | M | M |
| UPN29 | 15.02 | Mono | 10.6 | M | 8.91 | Oligo | 1.32 / 0.00 | U | M |
| UPN30 | 50.97 | Mono | 8.19 | M | 85.81 | Mono | 10.36 | M | M |
| UPN31 | 62.22 | Mono | 7.51 | M | 84.65 | Mono | 8.85 | M | M |
| UPN32 | 54.43 | Mono | 0 | U | 80.39 | Mono | 0 | U | I |
| UPN33 | 60.43 | Mono | 6.08 | M | 37.14 | Oligo | 6.64 / 6.14 | M | M |
| UPN34 | 62.78 | Mono | 8.78 | M | 86.02 | Mono | 8.66 | M | M |
| UPN35 | 70.44 | Mono | 5.07 | M | 56.15 | Oligo | 6.17 / 6.55 | M | M |
| UPN36 | 51.22 | Oligo | 7.77 | M | 71.56 | Oligo | 11.45 / 11.89 | M | M |
| UPN37 | 60.45 | Mono | 5.41 | M | 17.37 | Mono | 0 | U | M |
| UPN38 | 37.42 | Mono | 5.8 | M | 71.45 | Mono | 6.14 | M | M |
| UPN39 | 59.61 | Mono | 6.42 | M | 63.1 | Oligo | 7.96 / 0.44 | M | M |
| UPN40 | 72.47 | Mono | 3.04 | M | 89.64 | Mono | 3.96 | M | I |
| UPN41 | 68.66 | Mono | 5.07 | M | ND | ND | - | I | M |
| UPN42 | 63.94 | Mono | 8.45 | M | ND | ND | - | I | M |
| Abbreviations: *Mono* monoclonal, *Oligo* oligoclonal, *Poly* polyclonal, *ND* no clonality detected, *M* mutated, *U* unmutated, *I* inconclusive. | | | | | | | | | |

Table S2. Comparison of somatic hypermutation results between Sanger sequencing and the FR1 assay

| Somatic hypermutation | | Sanger sequencing | | |
| --- | --- | --- | --- | --- |
|  |  | SHM | No SHM | Inconclusive |
| FR1 assay | SHM | 22 | 0 | 1 |
|  | No SHM | 4 | 3 | 2 |
|  | Inconclusive | 9 | 0 | 1 |

Abbreviation: *SHM* somatic hypermutation.

Table S3. Comparison of somatic hypermutation results between Sanger sequencing and the Leader assay

| Somatic hypermutation | | Sanger sequencing | | |
| --- | --- | --- | --- | --- |
|  |  | SHM | No SHM | Inconclusive |
| Leader assay | SHM | 31 | 0 | 2 |
|  | No SHM | 1 | 3 | 1 |
|  | Inconclusive | 3 | 0 | 1 |

Abbreviation: *SHM* somatic hypermutation.
